# A fast solver for entropy-regularized damage transport

**DOI:** 10.1007/s00466-026-02816-4

**Published:** 2026-07-20

**Authors:** Matti Schneider

**Affiliations:** 1 Institute of Engineering Mathematics, University of Duisburg-Essen, Essen, Germany; 2 Center for Nanointegration Duisburg-Essen (CENIDE), Essen, Germany; 3 Fraunhofer Institute for Industrial Mathematics ITWM, Kaiserslautern, Germany

**Keywords:** Entropy-regularized damage transport, Isotropic damage model, Newton-bisection method, Well-posed model, Continuum damage mechanics

## Abstract

Convexification-based approaches to damage mechanics emerged recently as powerful alternatives to non-local or gradient-augmented damage models. Convexifying the incremental potentials leads to a damage-transport equation at material-point level, and a simple modification based on entropic regularization was recently shown to give rise to a continuum damage model which shows softening yet whose incremental potentials are strongly convex, ensuring well-posedness of the boundary-value problem and efficiency of computational resolution. These salient properties come at a price: The damage-transport equation, an advection-type equation, must be solved at each material point. The latter involves a discretization of the attainable damage states and seeks a large number of transition coefficients. The work at hand introduces a novel reformulation of the damage-transport equation as a one-dimensional root-finding problem. Once this problem is solved, the transition coefficients may be obtained in post-processing. We discuss the monotonicity and uniqueness properties of the one-dimensional function whose root is sought, provide a simple yet robust computational strategy for its resolution and study the efficiency of the approach in dedicated computational experiments.

## Introduction

The softening inherent to eventual failure of materials leads to difficulties in the numerical treatment. Local continuum damage models with softening [1–3] show erratic behavior in finite-element simulations, reflecting the underlying ill-posedness of the boundary-value problem at hand. Various modifications of damage (and fracture) models were proposed [4] to mitigate the consequences of the ill-posedness, including viscous regularization [5], non-local constitutive laws [6] and gradient-type models [7]. All of these strategies come with certain disadvantages, in particular they do not lead to well-posed models in the sense of Hadamard.

Recently, a different approach based on convex relaxation [8–10] was investigated (doubled words) see also Balzani et al. [11–14]. Attempts were made to construct continuum damage models which combine two advantageous properties: They show softening yet the incremental potentials are convex. Indeed, constructing convex damage models without softening is rather simple [15, 16]. For a simple isotropic damage model, Schwarz et al. [17] proposed to consider the cross-convex envelope of a rate-limited damage model [18]. However, the paper eventually falls short on considering the exact cross-convex envelope and rather introduces a computationally convenient approximation, called the emulated RVE. Lauff et al. [19] take the work of Schwarz et al. [17] as the starting point and calculate the cross-convex envelope exactly. This work clarifies the mathematical structure of the ensuing relaxed model, leading naturally to damage distributions instead of single-valued (“uniform”) damage variables. Interestingly, the evolution equation for the internal variables—the damage distributions—turns out to be a transport equation. In particular, convex relaxation naturally leads to a hyperbolic regularization, in contrast to the elliptic regularization inherent to gradient regularizations.

Unfortunately, convex relaxation is not sufficient to render the associated boundary-value problems well-posed: Although existence is ensured, uniqueness does not hold, in general. This observation is actually well-known in the literature and additional selection mechanisms are studied [20]. To rectify this shortcoming, Lauff et al. [21] introduced a delicate regularization term which makes the boundary-value problems uniquely solvable, its solutions depend continuously on the data, yet preserves the salient features of the original model: There is an elastic domain and, for each time step, the natural implicit scheme has discrete solutions.

### State of the art and contributions

The work at hand addresses a shortcoming of the entropy-regularized damage-transport model [21], summarized in Sect. 2.1: For each time step, the material model needs to resolve a constrained nonlinear optimization problem with a high number of degrees of freedom. The latter correspond to the number of attainable damage states required to parameterize the resulting damage distributions. Based on a careful analysis of the KKT conditions, we find that only the final effective degradation value needs to be found, and the sought degrees of freedom may be computed explicitly in a postprocessing step, see Sect. 2.2. In particular, only a single equation needs to be solved. However, just using a vanilla Newton method does not work robustly—due to the “change of variables”, the one-dimensional problem is severely nonlinear. As a remedy, we propose to use a Newton-bisection method, see Sect. 2.3 for details. We close the article with computational investigations, see Sect. 3.

## Solving regularized damage transport

### Regularized damage transport in a nutshell

The section at hand provides the essentials from the recent work [21], called Entropy-regularized Damage Transport (EDT) in the following, required for the article at hand. Working in an isothermal setting at small strains, we suppose that an elastic stiffness tensor $\mathbb {C}$ together with a damage-degradation function

2.1 $$\begin{aligned} f: \mathcal {Z} := [0,d_{\text {max}}) \rightarrow (0,1], \quad d\mapsto f(d), \end{aligned}$$

is given. The degradation function (2.1) is assumed to be strictly monotonically decreasing and to satisfy the boundary conditions

2.2 $$\begin{aligned} f(0) = 1 \quad \text {and} \quad \lim _{d \rightarrow d_{\text {max}}} f(d) = 0. \end{aligned}$$

Moreover, we assume the reciprocal degradation function

2.3 $$\begin{aligned} g: \mathcal {Z} \rightarrow [1,\infty ), \quad g(d) = \frac{1}{f(d)} \end{aligned}$$

to be strictly convex. The typical degradation function we have in mind is the exponential degradation [22]

2.4 $$\begin{aligned} f(d) = e^{-d} \quad \text {with reciprocal} \quad g(d) = e^d. \end{aligned}$$

The parameters for the model are completed by a damage-rate limitation $\dot{d}_{\text {max}}$ and two positive parameters *r* and λ which refer to the unregularized damage-initiation threshold and the regularization parameter.

For the current time step $t^n$ with time increment ▵t, we suppose that a number

2.5 $$\begin{aligned} d_1< d_2< \ldots < d_N, \quad d_k \in \mathcal {Z}, \end{aligned}$$

of discrete damage states are given, together with the associated mass or volume fractions of damage, described by a discrete probability distribution $\rho ^n$ associated to these damage states, i.e., values ${\rho ^n_k}$ which satisfy the simplex constraints

2.6 $$\begin{aligned} \rho ^n_k \ge 0 \quad \text {for all} \quad k=1,2,\ldots ,N \quad \text {and} \quad \sum _{k=1}^N \rho ^n_k = 1. \end{aligned}$$

The update to the subsequent time step with (suppressed) index n+1 is governed by *N* transition coefficients $\phi _k \in [0,1]$, s.t., a damage mass $(1-\phi ) \, \rho ^n_k$ remains at the damage state $d_k$, whereas an amount $\phi \, \rho ^n_k$ is shifted to the elevated damage state

2.7 $$\begin{aligned} d_k^+ = d_k + \triangle t \, \dot{d}_{\text {max}}. \end{aligned}$$

Here, we assume that the maximum damage $d_N^+$ does not exceed the maximum attainable damage $d_{\text {max}}$. Traditionally, a uniform discretization with increment $\triangle t \, \dot{d}_{\text {max}}$ is used in damage space.

Using the symbol $\boldsymbol{\upphi }$ to denote the collection of transition coefficients, the corresponding free energy reads

2.8 $$\begin{aligned} w(\boldsymbol{\varepsilon }, \rho ) = \bar{f}(\boldsymbol{\upphi }) \, w_0(\boldsymbol{\varepsilon }) \end{aligned}$$

with the undamaged energy

2.9 $$\begin{aligned} w_0(\boldsymbol{\varepsilon }) = \frac{1}{2} \, \boldsymbol{\varepsilon }: \mathbb {C}: \boldsymbol{\varepsilon }\end{aligned}$$

and the effective degradation function

2.10 $$\begin{aligned} \bar{f}(\boldsymbol{\upphi }) = \left[ \sum _{k=1}^N \rho ^n_k \left( (1-\phi _k) \, g(d_k) + \phi _k \, g(d_k^+)\right) \right] ^{-1}. \end{aligned}$$

The dissipation distance has the form

2.11 $$\begin{aligned} \mathcal {D}(\boldsymbol{\upphi }) \equiv \triangle t \, \dot{d}_{\text {max}}\sum _{k=1}^N \rho ^n_k \, (r\, \phi _k - \lambda \, \log (1-\phi _k)). \end{aligned}$$

From a mechanical point of view, the presented model can be interpreted as follows [17, 21]. Consider a rod comprising *N* segments of relative length $\rho ^n_k$. Each of the segments with index k=1,2,…,N is assumed to have a fixed damage state $d_k$ with degradation $f(d_k)$. In a single time step, each segment is allowed to damage further by switching a fraction $\phi _k$ of the segment to the advanced damage state $d_k^+ = d_k + \triangle t\,\dot{d}_{\text {max}}$. The resulting Young’s modulus of the rod is given by the Reuss model, i.e., the harmonic average (2.10). In turn, the switching leads to a dissipation, quantified by the expression (2.11). The dissipated energy consists of a linear term proportional to the fraction of switching $\phi _k$ and an additional logarithmic term which penalizes the switching. The latter term may be interpreted as a “sticking mechanism”.

We observe from Eq. (2.11) that the damage-rate limitation parameter $\dot{d}_{\text {max}}$ translates into a limitation for the dissipated energy during the time step at hand. Following the established route [23, 24], the time evolution of the prescribed model is governed by solving the time-incremental problem

2.12 $$\begin{aligned}  &   w(\boldsymbol{\varepsilon }, \rho (\boldsymbol{\upphi })) + \mathcal {D}(\boldsymbol{\upphi }) \equiv \bar{f}(\boldsymbol{\upphi }) \, w_0(\boldsymbol{\varepsilon }) \nonumber \\  &   \quad {+} \triangle t \, \dot{d}_{\text {max}}\sum _{k=1}^N \rho ^n_k \, (r\, \phi _k {-} \lambda \, \log (1{-}\phi _k)) \longrightarrow \min _{\boldsymbol{\upphi }\in [0,1]^N}. \end{aligned}$$

In the previous works [19, 21], these equations were solved by some sort of projected gradient method with adaptive step size, see Algorithm 3 below. In this way, the use of Lagrange multipliers could be avoided. Still, a nonlinear optimization problem with *N* independent unknowns needed to be solved.

We refer to the original work [21] for details about further aspects of computational mechanics, e.g., the material tangent.

### Re-casting the optimality conditions

We take a closer look at the optimization problem (2.12)

2.13 $$\begin{aligned} \bar{f} \, w_0 + \triangle t \, \dot{d}_{\text {max}}\sum _{k=1}^N \rho ^n_k \, (r\, \phi _k - \lambda \, \log (1-\phi _k)) \longrightarrow \min _{0 \le \phi _k < 1}, \end{aligned}$$

where we drop the dependence of the effective degradation $\bar{f}$ on the fractions $\phi _k$ and the dependence of the undamaged free energy (2.9) on the strain for notational brevity. To study the optimality conditions, we introduce the Lagrangian

2.14 $$\begin{aligned}  &   \mathcal {L}(\phi _1,\ldots ,\phi _n,\mu _1,\ldots ,\mu _n) = \bar{f} \, w_0 \nonumber \\  &   \quad {+} \triangle t \, \dot{d}_{\text {max}}\sum _{k=1}^N \rho ^n_k \, (r\, \phi _k {-} \lambda \, \log (1{-}\phi _k)) {-} \sum _{k=1}^N \mu _k \, \phi _k, \end{aligned}$$

where $\mu _k$ refers to the Lagrange multiplier associated to the inequality constraint $\phi _k \ge 0$. The upper bound on the volume fraction $\phi _k$ is implicitly enforced by the logarithmic barrier term. To proceed, we record the partial derivative [19, 21]

2.15 $$\begin{aligned} \frac{\partial \bar{f}}{\partial \phi _k} = - \bar{f}^{\,2} \, \rho ^n_k \, [g(d_k^+) - g(d_k)]. \end{aligned}$$

Then, the KKT conditions for the Lagrangian (2.14) read

2.16 $$\begin{aligned} {-} \bar{f}^{\,2} \, \rho ^n_k \, [g(d_k^+) - g(d_k)] \, w_0 + \triangle t \, \dot{d}_{\text {max}}\, \rho ^n_k \left( r {+} \frac{\lambda }{1-\phi _k} \right) {=} \mu _k \end{aligned}$$

as well as

2.17 $$\begin{aligned} \mu _k \ge 0, \quad \mu _k \, \phi _k = 0, \quad \phi _k \ge 0, \end{aligned}$$

for all k=1,2,…,N. For simplicity of exposition, we assume that the coefficients $\rho ^n_k$ are actually positive—otherwise there would be no contribution from the variable $\phi _k$. Multiplying the first equation (2.16) by the volume fraction $\phi _k$ and accounting for the complementarity constraints (2.17), we find that the volume fraction $\phi _k$ solves the equation

2.18 $$\begin{aligned} - \bar{f}^{\,2} \, [g(d_k^+) - g(d_k)] \, w_0 + \triangle t \, \dot{d}_{\text {max}}\, \left( r + \frac{\lambda }{1-\phi _k} \right) = 0 \end{aligned}$$

in case it is positive. Solving for the unknown $\phi _k$ therefore yields the expression

2.19 $$\begin{aligned} \phi _k = \left\{ \begin{array}{rl} \frac{ \bar{f}^{\,2} \, w_0 \, Q_k - r - \lambda }{\bar{f}^{\,2} \, w_0 \, Q_k - r}, &  \bar{f}^{\,2} \, w_0 \, Q_k \ge r + \lambda ,\\ 0,& \text {otherwise,} \end{array} \right. \end{aligned}$$

where we introduced the short-hand notation

2.20 $$\begin{aligned} Q_k = \frac{g(d_k^+) - g(d_k)}{\triangle t \, \dot{d}_{\text {max}}}. \end{aligned}$$

### Devising a robust numerical strategy

The starting point for the work at hand is the realization that the explicit formula (2.19) for the volume fraction $\phi _k$ may be considered as a *function* of the effective degradation function $\bar{f}$. Thus, as soon as the proper value $\bar{f}$ is known, the damage-volume fractions (2.19) are readily obtained.

As a summary of the previous section, the following two equations

2.21 $$\begin{aligned} \bar{f}&= \left[ \sum _{k=1}^N \rho ^n_k \left( (1-\phi _k) \, g(d_k) + \phi _k \, g(d_k^+)\right) \right] ^{-1}, \end{aligned}$$

2.22 $$\begin{aligned} \phi _k&= \frac{ \left\langle \bar{f}^{\,2} \, w_0 \, Q_k - r - \lambda \right\rangle _+}{\bar{f}^{\,2} \, w_0 \, Q_k - r} \end{aligned}$$

must be solved, where

2.23 $$\begin{aligned} \langle x \rangle _+ = \max (0,x), \quad x \in \mathbb {R}, \end{aligned}$$

stands for the Macauley bracket and we use the convention that the fraction (2.22) is identically zero in case the numerator vanishes.

**Fig. 1:**
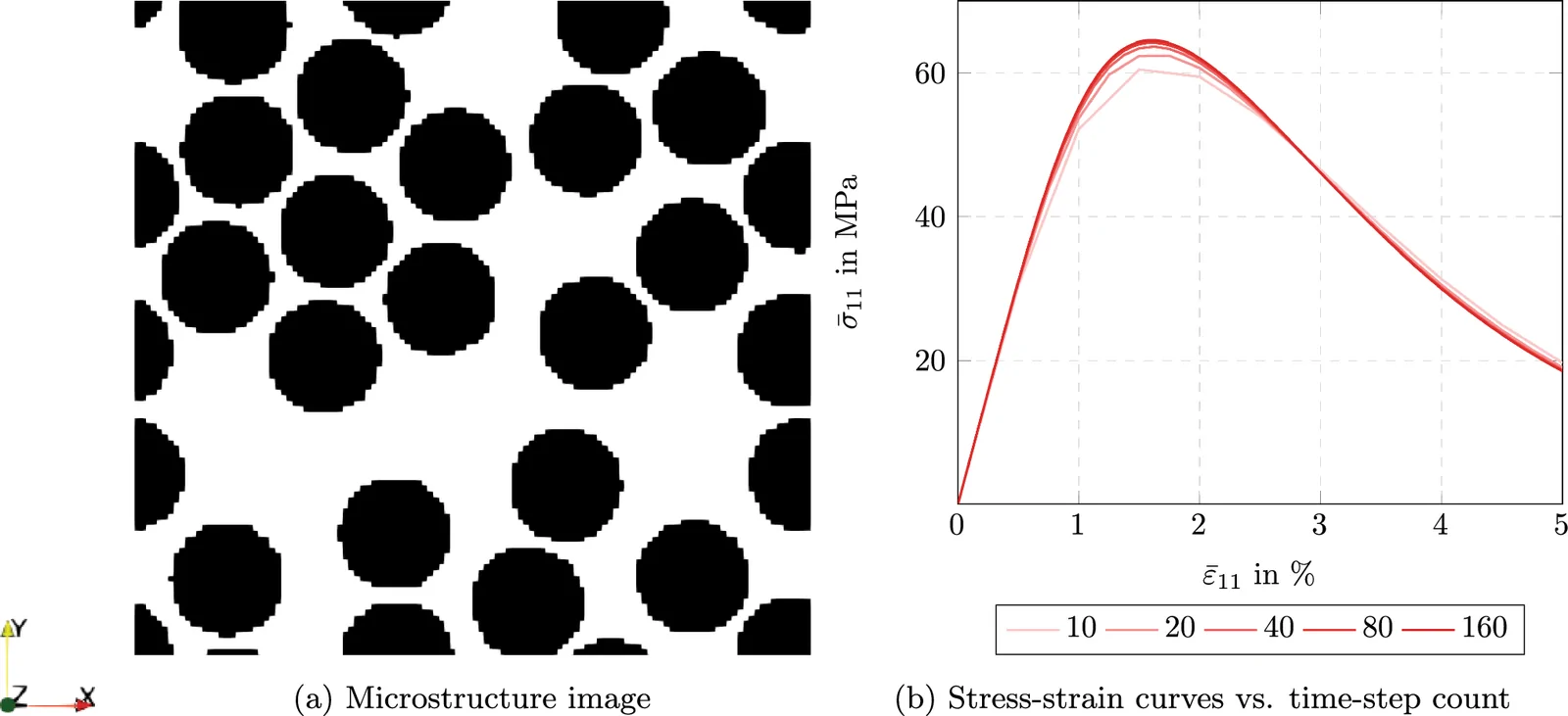
Considered microstructure and influence of the temporal resolution

Therefore, we consider Eq. (2.22) to define an explicit functional dependence of the volume fraction $\phi _k$ on the effective degradation $\bar{f}$, denoted by $\phi _k(\bar{f})$, and regard the relationship (2.21) as the equation to solve. Thus, we seek a root of the function

2.24 $$\begin{aligned}  &   F:[0,1] \rightarrow \mathbb {R}, \quad F(\bar{f}) = \bar{f} \nonumber \\  &   \quad - \left[ \sum _{k=1}^N \rho ^n_k \left( (1-\phi _k{(\bar{f})}) \, g(d_k) + \phi _k{(\bar{f})} \, g(d_k^+)\right) \right] ^{-1}. \end{aligned}$$

This is just a one-dimensional function and we strive to apply Newton’s method. Therefore, we compute the derivative

2.25 $$\begin{aligned}  &   F'(\bar{f}) {=} 1 {+} \left[ \sum _{k=1}^N \rho ^n_k \left( (1{-}\phi _k{(\bar{f})}) \, g(d_k) {+} \phi _k{(\bar{f})} \, g(d_k^+)\right) \right] ^{-2} \nonumber \\  &   \quad \sum _{k=1}^N \rho ^n_k \, (g(d_k^+) - g(d_k)) \frac{\partial \phi _k{(\bar{f})}}{\partial \bar{f}} \end{aligned}$$

with the sensitivity

2.26 $$\begin{aligned} \frac{\partial \phi _k{(\bar{f})}}{\partial \bar{f}} = \left\{ \begin{array}{rl} \frac{ 2\bar{f} \, w_0 \, Q_k \,\lambda }{(\bar{f}^{\,2} \, w_0 \, Q_k - r)^2}, &  \bar{f}^{\,2} \, w_0 \, Q_k \ge r + \lambda ,\\ 0,& \text {otherwise.} \end{array} \right. \end{aligned}$$

Notice that all the terms in the sum (2.25) are non-negative. Therefore, we find the elementary lower bound

2.27 $$\begin{aligned} F'(\bar{f}) \ge 1 \end{aligned}$$

on the slope of the function (2.24). In particular, there is at most one root of the function (2.24).

**Fig. 2:**
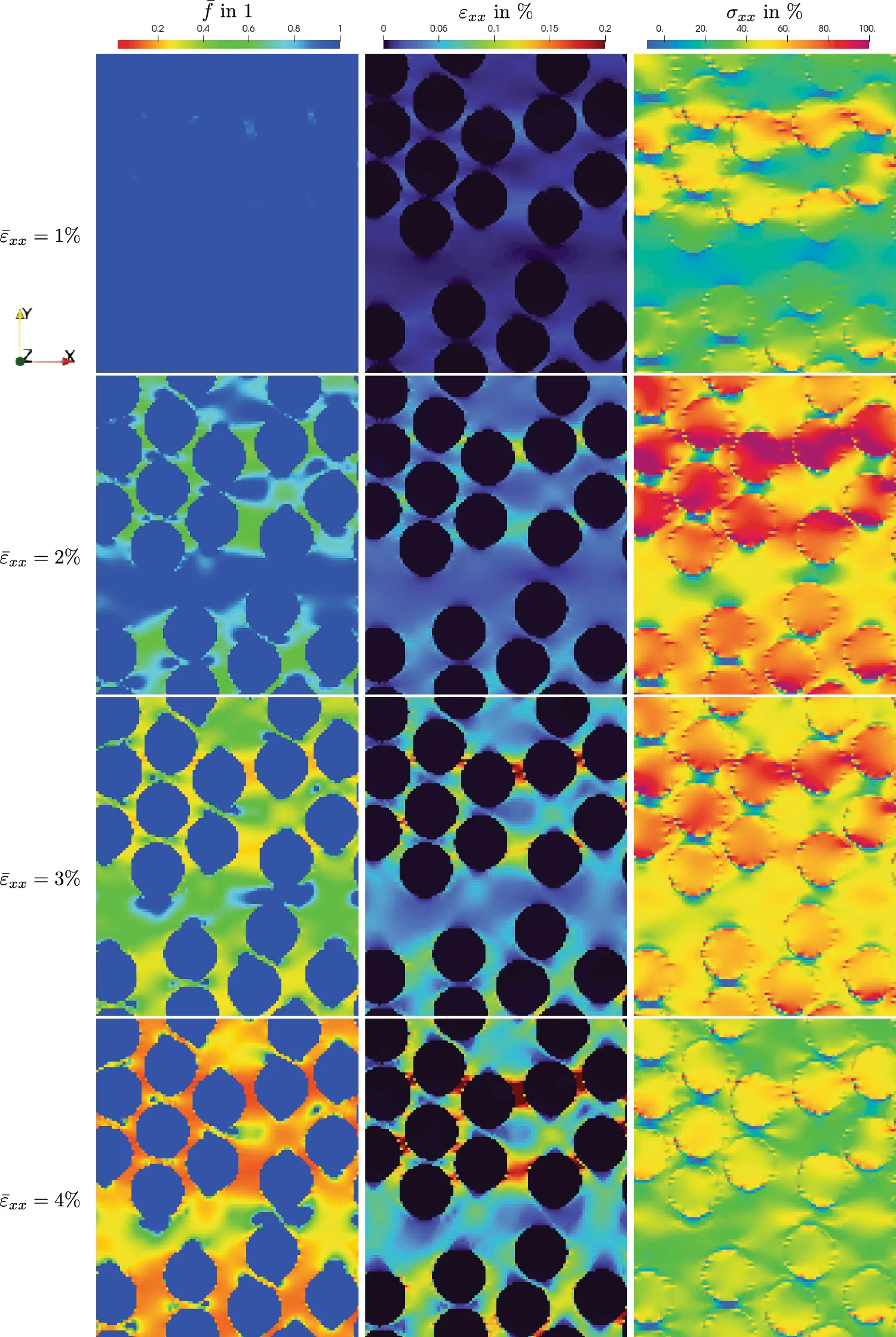
Local solution fields at different load levels resolving the curve shown in Fig. 1b

Although Newton’s method is known to converge quickly close to the root, we wish to empower the global convergence properties of the method by injecting some physical knowledge. In view of Eq. (2.21), the lower-upper bounds $0 \le \phi _k < 1$ and the monotonicity of the reciprocal degradation (2.3) lead to the elementary bounds

2.28 $$\begin{aligned} \sum _{k=1}^N \rho ^n_k \, g(d_k)  &   \le \sum _{k=1}^N \rho ^n_k \left( (1{-}\phi _k{(\bar{f})}) \, g(d_k) {+} \phi _k{(\bar{f})} \, g(d_k^+)\right) \nonumber \\  &   < \sum _{k=1}^N \rho ^n_k \, g(d_k^+). \end{aligned}$$

Therefore, we find the bracketing bounds

2.29 $$\begin{aligned} \bar{f}_- \equiv \left[ \sum _{k=1}^N \rho ^n_k \, g(d_k^+) \right] ^{-1} < \bar{f} \le \bar{f}_+ \equiv \left[ \sum _{k=1}^N \rho ^n_k \, g(d_k) \right] ^{-1}. \end{aligned}$$

The corresponding values of the function (2.24) satisfy the relations

2.30 $$\begin{aligned} F(\bar{f}_-) < 0 \quad \text {and} \quad F(\bar{f}_+) \ge 0. \end{aligned}$$

The value $\bar{f}_+$ may thus serve as an “elastic predictor”.

A naive implementation of Newton’s method for finding a root of the function (2.24) did not convince: For large time steps, the convergence behavior turned out to be erratic. Therefore, we changed to a combination of Newton’s method with the classical bisection method, see Press et al. [25, §9.4], for instance. Such a strategy combines the rapid local convergence of Newton’s method with the strong global convergence guarantees of the bisection method. A pseudo-code of the resulting algorithm is recorded in Algorithm 2. Compared to the reference [25, §9.4], we included more early-exit strategies to accelerate the scheme. Indeed, the original algorithm [25, §9.4] calls the function (2.24) at least thrice and presupposes strict bounds to be given. Such a strategy appears inefficient for the elastic domain, i.e., before the onset of damage, or in case of small time-step size. We therefore start with a single function evaluation to permit an early exit. Then, we make a single Newton step to facilitate fast convergence for a small time step. Only in case the sign of the objective function does not change, we resort to the lower bound (2.29). Indeed, a change in the sign of the objective function at the bracketing values is required to start the bisection.

For completeness, we also record the original algorithm [21] in Algorithm 3.

**Algorithm 1:**
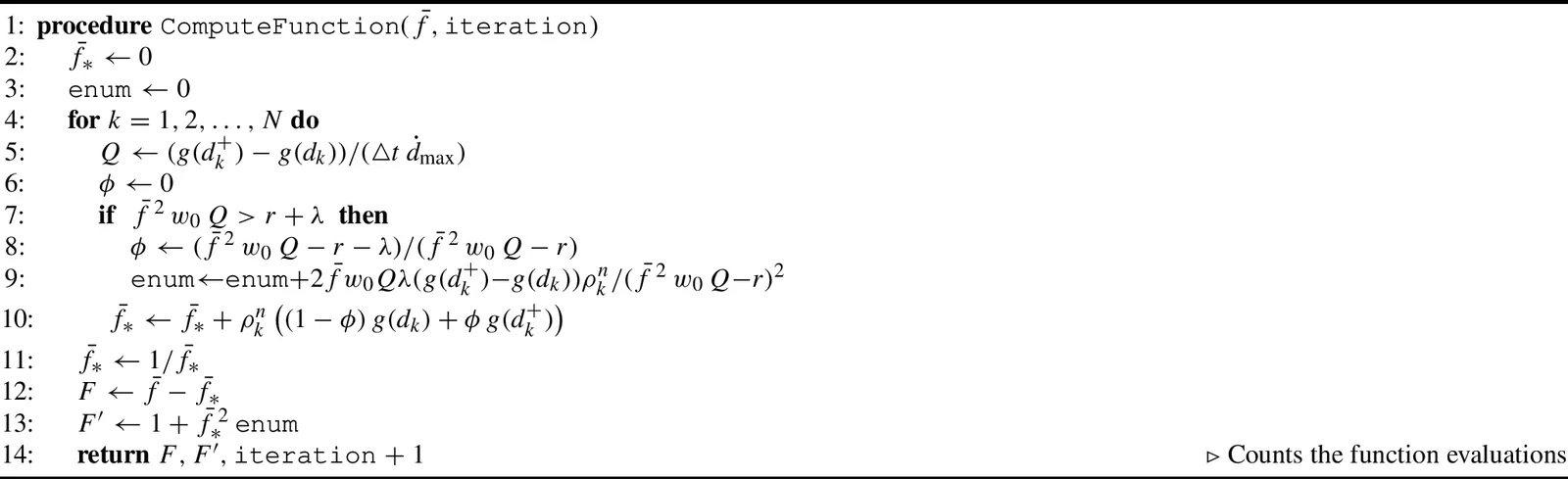
Computing the function (2.24) and its derivative (2.25)

**Algorithm 2:**
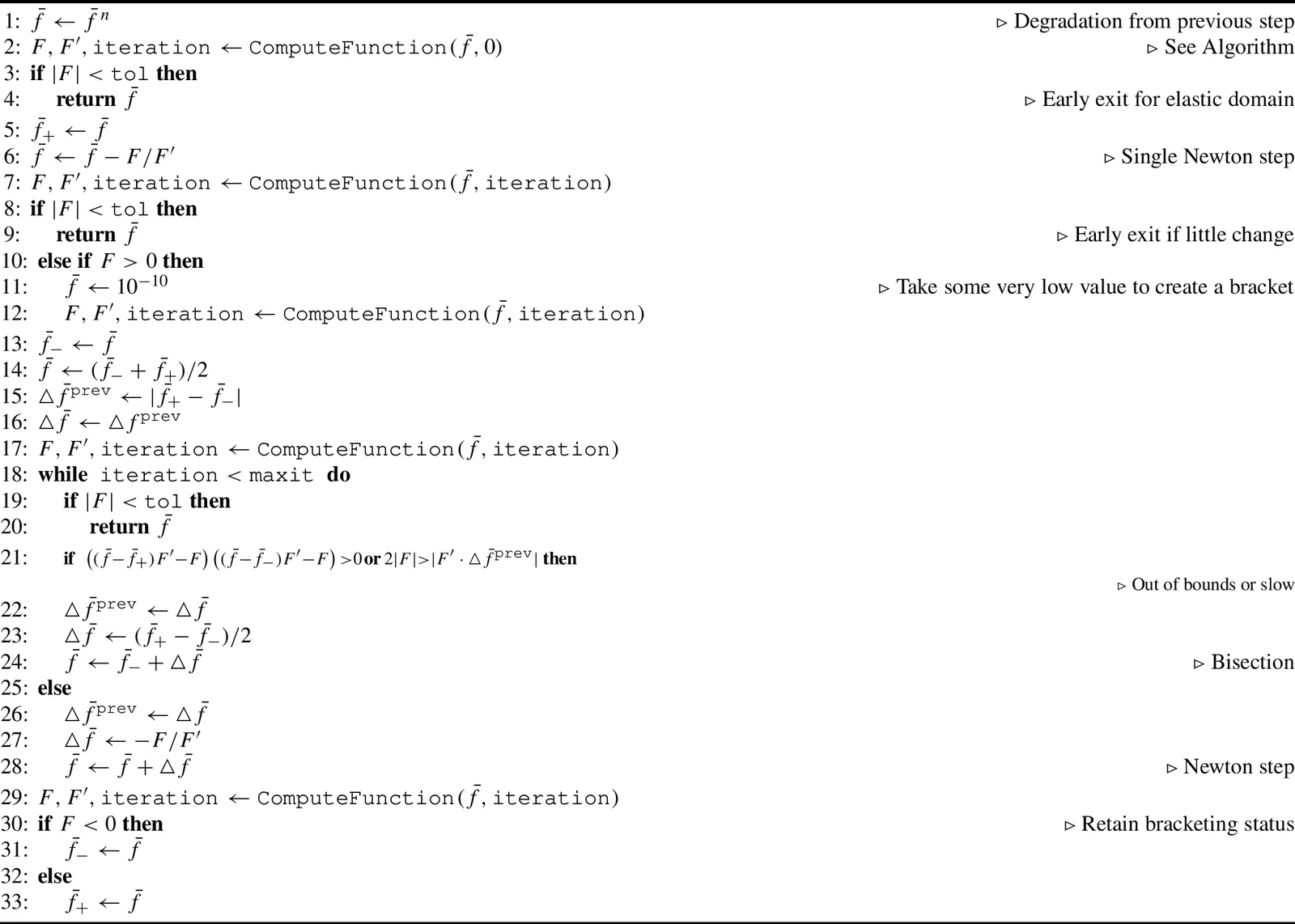
Newton-bisection for regularized damage transport $(w_0;\texttt {maxit}, \texttt {tol})$, the “$\bar{f}$-solver”

**Algorithm 3:**
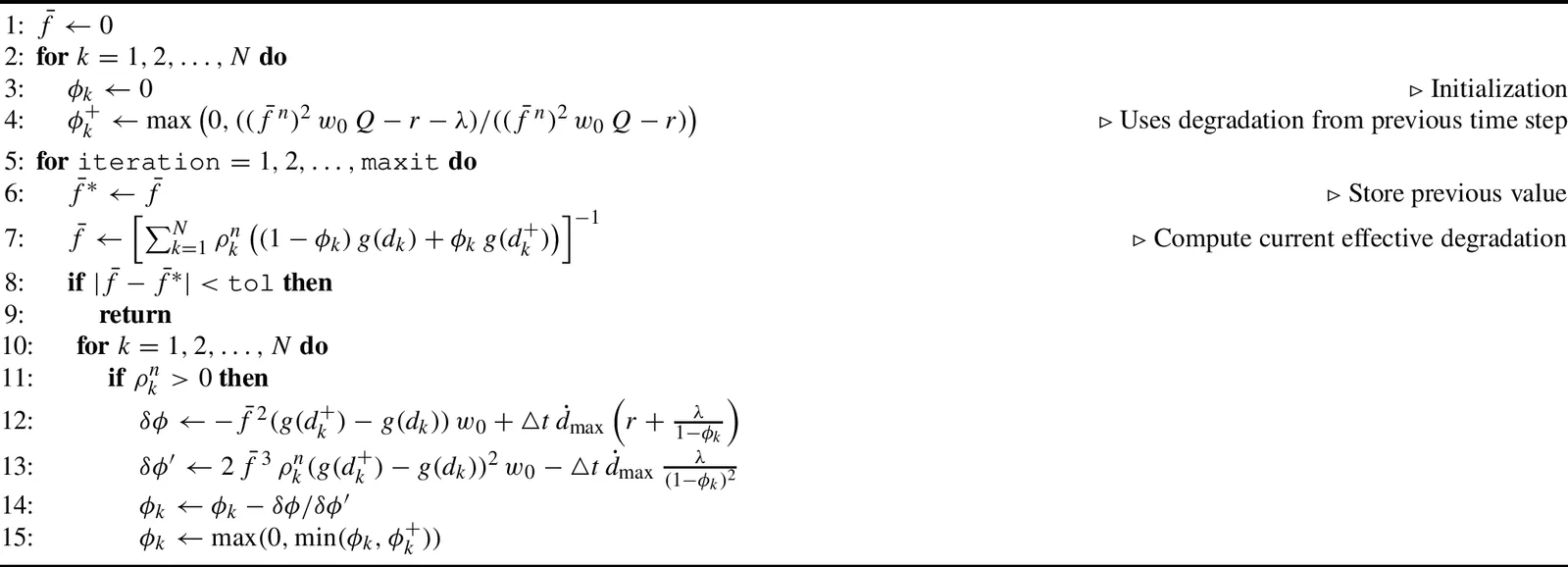
Diagonal Newton-type method [21] $(w_0;\texttt {maxit}, \texttt {tol})$, the “ϕ-solver”

## Computational experiments

To study the performance of the introduced solver, we investigate an example from micromechanics. More precisely, we consider a continuously reinforced fiber composite. The microstructure, comprising 50% volume fraction of E-glass fibers, is shown in Fig. 1a, with 22 circular inclusions and a resolution of 120×120 pixels.

**Fig. 3:**
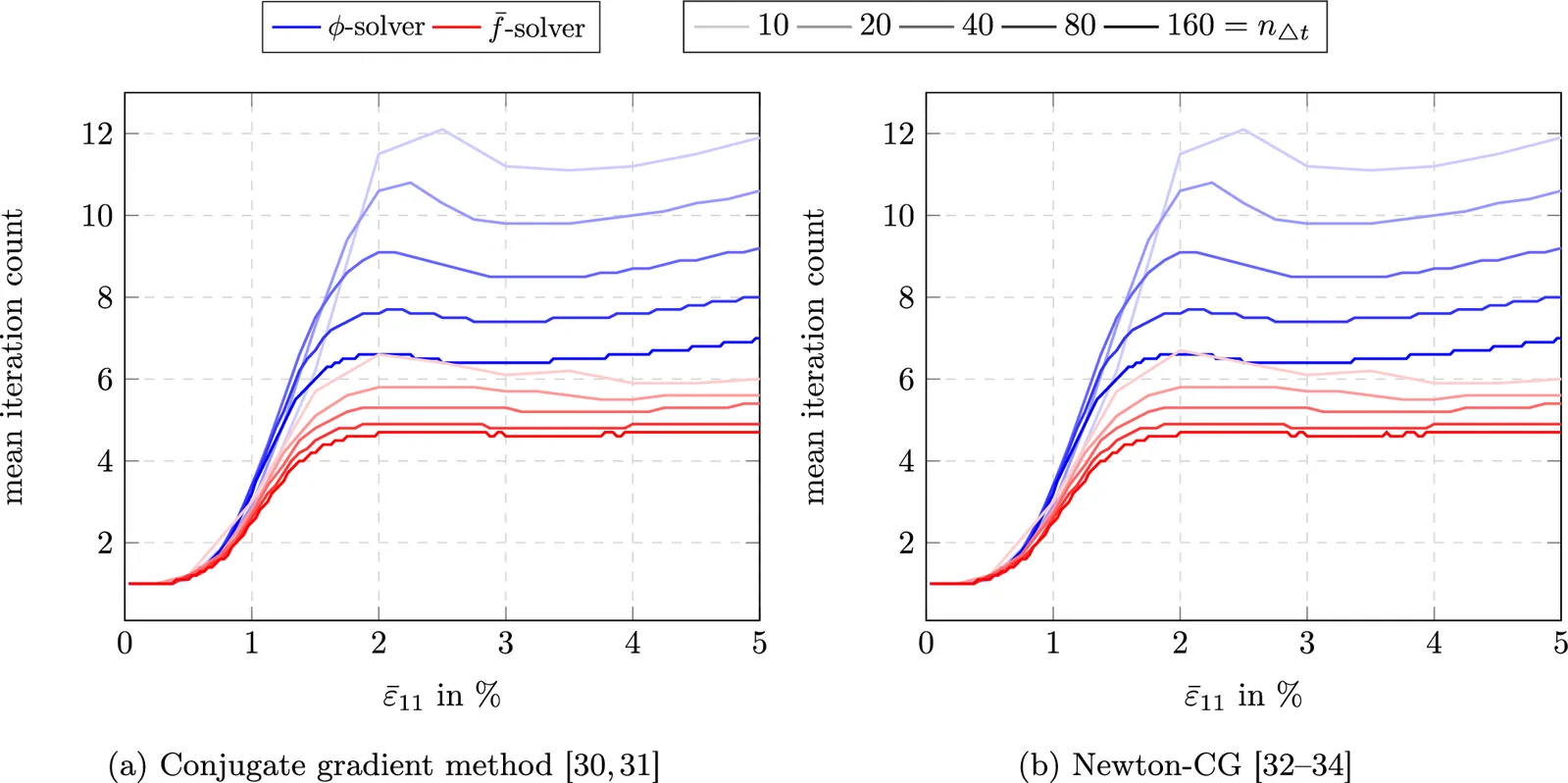
Mean iteration count at material level for different microstructure solvers

We furnish the inclusions with the isotropic elastic parameters of E-glass: E=72 GPa and ν=0.22. For the polyamide matrix, we use an EDT model [21], as discussed in Sect. 2.1. We use the exponential degradation function (2.4) and utilize the parameters E=2.1 GPa, ν=0.3, r=0.45 MPa, $\dot{d}_{\text {max}}= 4/s$, λ=0.05 MPa. The problem is discretized on a staggered grid [26] and solved via an in-house FFT-based computational micromechanics solver [27, 28]. We use the convergence criterion described in Schneider [29, §3.6] with a tolerance of $10^{-5}$. We consider two different solvers: Nonlinear CG [30, 31] and Newton-CG [32–34], where CG stands for the Conjugate Gradient method [35–37]. The Newton-CG method needs little introduction, yet its use in FFT-based micromechanics is limited due to the strong memory demand of storing the material tangents locally and the overhead induced by the four vectors required in the (linear) CG method [38, 39]. More popular in FFT-based micromechanics are low-memory solvers which, however, require evaluating the constitutive law at each iteration. The nonlinear CG method [30, 31] provides a good balance between speed and stability for inelastic constitutive laws.

We consider uniaxial extension in *x*-direction with a strain rate of $\dot{{{\,\mathrm{\varepsilon }\,}}}_{xx} = 5\%/s$ applied for 1 s. The resulting stress–strain curve is shown in Fig. 1b for a varying number of equidistant time steps. We observe that a low number of time steps does not reproduce the stress–strain curve with high fidelity. At least 80 time steps appear to resolve the stress–strain curve adequately. Yet, the low time-step count is interesting to study the behavior of the material-point solvers for large time steps. It should be mentioned that we use an internal tolerance of $10^{-10}$ for the material models. In particular, this level of fidelity leads to a convergence behavior of the microstructure-scale solver that is independent of the solver used for the material model.

To get an impression about the local fields during the loading, we refer to Fig. 2.

We observe that high strains build up in the regions where the inclusions are close and where they form “chains” in *x*-direction. These are also the regions where the local damage degradation is the most severe. Moreover, there are “shadow” regions at the inclusions where little to no damage degradation occurs. We also observe a medium degradation between the inclusions. Taking a closer look at the stress field, we realize that the highest stresses are observed at around 2% of strain loading, i.e., the microstructure experiences a local softening as well. This observation concurs with the expectations inferred from the stress–strain curve, see Fig. 1b .

The average iteration counts of the material model for the different solvers are recorded in Fig. 3. The solver presented in the work [21] and described in Algorithm 3 is shown in blue. The novel solver introduced in the work at hand and specified in Algorithm 2 is shown in red. The different time-step counts are indicated by different shades of the color under consideration. Moreover, the two different microstructure-scale solvers, nonlinear CG and Newton-CG, are shown in the two Fig. 3a and b respectively.

**Table 1:** Runtime on the microstructure shown in Fig. 1a

Method	10	20	40	80	160
CG & ϕ-solver	20.1 s	30.35 s	61.9 s	182.3 s	290.3 s
CG & $\bar{f}$-solver	8.9 s	18.0 s	33.9 s	93.6 s	123.7 s
Newton-CG & ϕ-solver	9.6 s	11.4 s	18.3 s	33.3 s	69.4 s
Newton-CG & $\bar{f}$-solver	8.5 s	11.2 s	16.6 s	27.0 s	56.9 s

We discuss the nonlinear CG Fig. 3a first. For all considered scenarios, only a single iteration is required for the initial elastic phase. For higher levels of loading, the mean iteration count increases up to roughly 2 to 2.5% of strain. Then, there is a slight dip, followed by another increase in iteration count. We observe that the mean iteration count decreases for decreasing time-step size. This phenomenon reflects the better initial guess provided by using smaller time steps.

For ten load steps and the CG method, the ϕ-solver requires between 1.14 and 12.06 iterations on average with a mean of 9.03 iterations. In contrast, for the largest time-step count of 160, between 1 and 6.96 iterations are required, with an average of 5.39. Thus, we also observe that the coarsest time resolution does not resolve the initial elastic behavior sufficiently. If Newton-CG is used as the microstructure-scale solver, there is little difference in the iteration statistics: Between 1.13 and 12.10 iterations are required on average with a mean of 9.03 iterations for ten load steps. Similar minuscule differences are observed for the other cases.

Taking a look at the $\bar{f}$-solver for ten load steps, between 1.20 and 6.65 iterations are necessary on average, with a mean of 5.30 iterations. For 160 load steps, between one and 4.68 iterations are taken on average, with an average of 3.88. Changing the microscale solver leads to a similar minimal change to the considered iteration counts.

All in all, we observe that the $\bar{f}$-solver introduced in the work at hand and specified in Algorithm 2 leads to an improvement by a factor of almost two compared to the previously developed Algorithm 3 in terms of iteration count. As the complexity of each individual iteration is comparable, a similar improvement in the material evaluation time emerges.

However, the full runtime is particularly interesting in practice. Therefore, we recorded the total runtime in Table 1, measured on an Ubuntu Laptop computer with 32 GiB of RAM and 12 i7-1355U cores, running on six (physical threads) with an OpenMP parallelization and FFTW [40]. We refer to the relevant works [27–29] for more details on the computational homogenization framework. For the CG solver, we observe an improvement roughly by a factor of two. As already mentioned, the iteration counts of the homogenization solver are identical or differ by at most a single iteration, so the differences in runtime are primarily caused by the material runtime. In any case, we observe a consistent and noticeable improvement in the runtimes as a result of the superior solver technology.

In addition to the CG solver, also the runtimes for the Newton-CG solver is detailed in Table 1. We observe that the runtime for Newton-CG improves upon the CG runtimes by factors of roughly two and three. This result does not come as a surprise: Newton-CG utilizes a number of applications of the elastic tangent material, thus side-stepping the costly evaluation of the inelastic material law. Still, there is a consistent improvement of the runtime if the novel $\bar{f}$-solver is used. For 160 time steps, the improvement is roughly 20% in runtime. For the other time-step counts, the improvement is similar except for the $n_{\triangle t} = 20$ case. Taking a closer look at the material runtime, we observe the material-evaluation runtime emerges as 1.5 s for the $\bar{f}$-solver compared to 2.5 s for the ϕ-solver. So, there is improvement in the runtime, yet the material runtime represents a small fraction of the total runtime, only. In contrast, for 160 time steps, 41.6 s are spent on evaluating the materials and the ϕ-solver, whereas 24.8 s are required for evaluating the constitutive laws for the $\bar{f}$-solver. Thus, the material evaluation takes up a larger fraction of the runtime for the more finely resolved examples.

## Conclusions

The work at hand contributes to the convexified damage models [19, 21] recently introduced into damage mechanics. More precisely, we took a critical look at the evaluation of the constitutive law for the entropy-regularized damage-transport model introduced in Lauff et al. [21]. This isotropic damage model permits to represent softening in the stress–strain curve, yet leads to a well-posed mechanical boundary value problem at every time step. The latter salient property is ensured by a uniformly positive definite material tangent inside the material model. The price to pay for these advantageous numerical properties is the large number of internal variables that need to be considered. In fact, the continuum model is based on damage distributions, in the first place, and, in principle, infinitely many degrees of freedom are required to describe an arbitrary damage distribution. In practice, only a finite number of degrees of freedom of the internal variables is considered. Still, a potentially large system of (nonlinear) equations must be resolved at each time step for the model at hand.

The article at hand is based on the observation that only a single scalar equation needs to be solved at each time step, and the updated values of the internal variables may be obtained by a simple post-processing. In hindsight, this revelation might be considered less surprising, as an isotropic damage model should lead to a single equation to solve. Yet, exploiting the one-dimensional formulation turned out to be more complicated than expected: The formulation with many degrees of freedom appears to be simpler and less nonlinear than its one-dimensional cousin. In particular, a simple 1D-Newton method turned out to lack robustness for large time steps. As a remedy, we proposed to combine the Newton scheme with a bisection method. The resulting algorithm combines the robustness of the bisection method with the high accuracy achievable with Newton’s method. Dedicated computational examples showed the advantages of the novel material-point solver.

This work is part of a broader research effort establishing a well-posed continuum damage model with salient mathematical and numerical properties. Providing a simple, robust and fast solver is a key aspect required for making this groundbreaking class of damage models ready for industrial use. An important next step is to extend the model to handle non-equispaced time steps and corresponding damage values.

## Data Availability

The data that support the findings of this study are available from the corresponding author upon reasonable request.

## References

[1] Lemaitre J (1996) A course on damage mechanics, 2nd edn. Springer

[2] Voyiadjis GZ, Kattan PI (2010) Advances in damage mechanics: metals and metal matrix composites with an introduction to fabric tensors, 2nd edn. Elsevier Science

[3] Murakami S (2012) Continuum damage mechanics, volume 185 of solid mechanics and its applications. Springer Netherlands, Dordrecht

[4] de Borst R, Sluys LJ, Muhlhaus H-B, Pamin J (1993) Fundamental issues in finite element analyses of localization of deformation. Eng Comput 10(2):99–121

[5] Needleman A (1988) Material rate dependence and mesh sensitivity in localization problems. Comput Methods Appl Mech Eng 67(1):69–85

[6] Pijaudier-Cabot G, Bažant ZP (1987) Nonlocal damage theory. J Eng Mech 113(10):1512–1533

[7] Peerlings RHJ, de Borst R, Brekelmans WAM, De Vree JHP (1996) Gradient enhanced damage for quasi-brittle materials. Int J Numer Methods Eng 39(19):3391–3403

[8] Dacorogna B (1982) Quasiconvexity and relaxation of nonconvex problems in the calculus of variations. J Funct Anal 46(1):102–118

[9] Carstensen C, Hackl K, Mielke A (2002) Non-convex potentials and microstructures in finite-strain plasticity. Proc R Soc Lond Ser A Math Phys Eng Sci 458(2018):299–317

[10] Mielke A (2003) Evolution of rate-independent inelasticity with microstructure using relaxation and young measures. In: Miehe C (ed) IUTAM symposium on computational mechanics of solid materials at large strains, vol 108. Springer Netherlands, Dordrecht, pp 33–44

[11] Balzani D, Ortiz M (2012) Relaxed incremental variational formulation for damage at large strains with application to fiber-reinforced materials and materials with truss-like microstructures. Int J Numer Methods Eng 92(6):551–570

[12] Balzani D, Köhler M, Neumeier T, Peter MA, Peterseim D (2024) Multidimensional rank-one convexification of incremental damage models at finite strains. Comput Mech 73(1):27–47

[13] Köhler M, Neumeier T, Peter MA, Peterseim D, Balzani D (2024) Hierarchical rank-one sequence convexification for the relaxation of variational problems with microstructures. Comput Methods Appl Mech Eng 432:117321

[14] Melchior J, Köhler M, Balzani D (2026) Variationally consistent microstructure evolution and microsphere-based reconvexification for damage with application to arterial tissues. Mech Mater 212:105495

[15] Görthofer J, Schneider M, Hrymak A, Böhlke T (2022) A convex anisotropic damage model based on the compliance tensor. Int J Damage Mech 31(1):43–86

[16] Magino N, Köbler J, Andrä H, Welschinger F, Müller R, Schneider M (2022) A multiscale high-cycle fatigue-damage model for the stiffness degradation of fiber-reinforced materials based on a mixed variational framework. Comput Methods Appl Mech Eng 388:114198

[17] Schwarz S, Junker P, Hackl K (2021) Variational regularization of damage models based on the emulated RVE. Continuum Mech Thermodyn 33(1):69–95

[18] Allix O (2013) The bounded rate concept: a framework to deal with objective failure predictions in dynamic within a local constitutive model. Int J Damage Mech 22(6):808–828

[19] Lauff C, Schneider M, Böhlke T (2026) Numerically robust local continuum damage models with softening response via convex relaxation. Comput Methods Appl Mech Eng 452:118759

[20] Kohn RV, Müller S (1992) Relaxation and regularization of nonconvex variational problems. Rendiconti del Seminario Matematico e Fisico di Milano 62:89–113

[21] Lauff C, Schlichting A, Schneider M, Böhlke T (2026) A well-defined local continuum damage model with softening via convexification and entropic regularization. Submitted, pp 1–43

[22] Dimitrijevic BJ, Hackl K (2011) A regularization framework for damage-plasticity models via gradient enhancement of the free energy. Int J Numer Methods Biomed Eng 27(8):1199–1210

[23] Ortiz M, Stainier L (1999) The variational formulation of viscoplastic constitutive updates. Comput Methods Appl Mech Eng 171:419–444

[24] Miehe C (2002) Strain-driven homogenization of inelastic microstructures and composites based on an incremental variational formulation. Int J Numer Methods Eng 55:1285–1322

[25] Press WH, Teukolsky SA, Vetterling WT, Flannery BP (1992) Numerical recipes in C, 2nd edn. Cambridge University Press, Cambridge

[26] Schneider M, Ospald F, Kabel M (2016) Computational homogenization of elasticity on a staggered grid. Int J Numer Methods Eng 105(9):693–720

[27] Schneider M (2019) On the Barzilai-Borwein basic scheme in FFT-based computational homogenization. Int J Numer Methods Eng 118(8):482–494

[28] Schneider M (2020) Lippmann-Schwinger solvers for the computational homogenization of materials with pores. Int J Numer Methods Eng 121(22):5017–5041

[29] Schneider M (2021) A review of non-linear FFT-based computational homogenization methods. Acta Mech 232:2051–2100

[30] Schneider M (2020) A dynamical view of nonlinear conjugate gradient methods with applications to FFT-based computational micromechanics. Comput Mech 66:239–257

[31] Schneider M (2017) An FFT-based fast gradient method for elastic and inelastic unit cell homogenization problems. Comput Methods Appl Mech Eng 315:846–866

[32] Gélébart L, Mondon-Cancel R (2013) Non-linear extension of FFT-based methods accelerated by conjugate gradients to evaluate the mechanical behavior of composite materials. Comput Mater Sci 77:430–439

[33] Kabel M, Böhlke T, Schneider M (2014) Efficient fixed point and Newton-Krylov solvers for FFT-based homogenization of elasticity at large deformations. Comput Mech 54(6):1497–1514

[34] Wicht D, Schneider M, Böhlke T (2020) On Quasi-Newton methods in FFT-based micromechanics. Int J Numer Methods Eng 121(8):1665–1694

[35] Hestenes M, Stiefel E (1952) Methods of conjugate gradients for solving linear systems. J Res Natl Bur Stand 49:409–436

[36] Fletcher R, Reeves C (1964) Function minimization by conjugate gradients. Comput J 7:149–154

[37] Dai YH, Yuan Y (1999) A nonlinear conjugate gradient method with a strong global convergence property. SIAM J Optim 10(1):177–182

[38] Zeman J, Vondřejc J, Novák J, Marek I (2010) Accelerating a FFT-based solver for numerical homogenization of periodic media by conjugate gradients. J Comput Phys 229(21):8065–8071

[39] Brisard S, Dormieux L (2010) FFT-based methods for the mechanics of composites: a general variational framework. Comput Mater Sci 49(3):663–671

[40] Frigo M, Johnson SG (2005) The design and implementation of FFTW3. Proc IEEE 93:216–231

